# The mitochondrial genome of hydrothermal vent barnacle *Eochionelasmus coreana* (Cirripedia: Thoracica) from the Indian Ocean

**DOI:** 10.1080/23802359.2020.1851153

**Published:** 2021-03-15

**Authors:** Won-Kyung Lee, Benny K. K. Chan, Se-Jong Ju, Dongsung Kim, Se-Joo Kim

**Affiliations:** a Genome Editing Research Center, Korea Research Institute of Bioscience and Biotechnology, Daejeon, Korea; b Biodiversity Research Center, Academia Sinica, Taipei, Taiwan; c Global Ocean Research Center, Korea Institute of Ocean Science and Technology, Busan, Korea; d Marine Biology Major, University of Science & Technology, Daejeon, Korea; e Marine Ecosystem Research Center, Korea Institute of Ocean Science and Technology, Busan, Korea

**Keywords:** *Eochionelasmus coreana*, hydrothermal vent barnacle, Solitaire vent field, Indian Ocean, mitochondrial genome

## Abstract

Balanomorph *Eochionelasmus* species are hydrothermal vent endemic barnacles. In the genus *Eochionelasmus*, three species are known to date and they distribute at three different vent fields in Pacific and Indian Oceans, *E. ohtai* in the Southwest Pacific Ocean, *E. paquensis* in the East Pacific Ocean, and *E. coreana* in the Indian Ocean. Therefore, *Eochionelasmus* species are considered to be a meaningful model taxon to elucidate the evolutionary history of vent organism in relation to geotectonic events. Here, we characterized the partial mitogenome of a newly described vent barnacle *Eochionelasmus coreana* Chan et al., 2020 from the Solitaire vent field in the Indian Ocean. The length of mitogenome was 16,804 bp with 64.0% AT content. Its gene content and organization was identical to those of *E. ohtai*. There was one significant part in the mitogenome of *E. coreana*, which was a long intergenic region over 2 kb found between tRNA^Pro^ and tRNA^Thr^. The phylogenetic tree suggested the monophyly of *E. ohtai* and *E. coreana* with high supporting values. In the future, additional mitogenome analysis of the last *Eochionelasmus* species, *E. paquensis*, could expand our understanding about the speciation and global distribution of *Eochionelasmus* species.

Balanomorphs (acorn barnacles) are morphologically highly diversified and they form the largest thoracican suborder with over 1000 species which are commonly found in various marine habitats all over the world (Martin and Davis 2001; Ahyong et al. 2011). Among them, the genus *Eochionelasmus*, which is characterized by six wall plates and multiple whorls of imbricating plates, is considered the sole balanomorph genus restrictively living in hydrothermal vent environments (Yamaguchi and Newman 1990; Yamaguchi et al. 1997a, 1997b; Chan et al. 2020). Interestingly, three valid species within *Eochionelasmus* have been found at three different vent fields in the Pacific and Indian Oceans, *E. ohtai* in the Southwest Pacific Ocean, *E. paquensis* in the East Pacific Ocean, and *E. coreana* in the Indian Ocean (Yamaguchi et al. 1997a, 1997b; Chan et al. 2020). Therefore, *Eochionelasmus* is considered to be a good model taxon to understand the relationship between the distribution of hydrothermal vent organisms and geotectonic events on earth. In this study, we characterized the second mitochondrial genome (mitogenome) of *Eochionelasmus*, and compared the mitogenomes of two *Eochionelasmus* species, *E. ohtai* and *E. coreana*.

*Eochionelasmus coreana* specimens were collected in June 2018 from the Solitaire vent fields, Indian Ocean (19° 33′ 39″S, 65° 137 50′ 89″E; water depth: 2625 m) using TV-guided grab sampler. For mitogenome analysis in this study, we used the paratype specimen of *E. coreana* (Chan et al. 2020), which is deposited at the Biodiversity Research Museum, Academia Sinica, Taiwan (ASIZCR-000433). Genomic DNA extraction and mitochondrial DNA amplification were performed according to the method of Kim et al. (2018). Library construction and sequencing were carried out by Macrogen Service (Macrogen, Seoul, Korea) using Illumina NovaSeq sequencing platform (Illumina, San Diego, CA). The raw data were assembled using NOVOPlasty 2.7.2 (Dierckxsens et al. 2017) and further corrected using Geneious Prime (Biomatters, Auckland, New Zealand). The assembled mitogenome was annotated using MITOS (Bernt et al. 2013) and adjusted manually comparing the genome with other barnacles. After the annotation, we found a long intergenic region with repetitive and ambiguous sequences between tRNA^Pro^ and tRNA^Thr^. To determine this region, we performed polymerase chain reaction and Sanger sequencing using the newly designed primers (EC_ND4L_F: 5′-CCAATCCCGAGGTAAATCTC-3′; EC_ND6_R2: 5′-GTTGATCATAGCCTAGGAGG-3′). As a result, it was assumed to be approximately 2 kb by visualization of gel electrophoresis, but we could not confirm the complete sequence information.

The mitogenome of *E. coreana* was incompletely described with 16,804 bp in length (GenBank accession no. MT491209; 63.9% AT content), excluding ambiguous sequences between tRNA^Pro^ and tRNA^Thr^. It contained 13 protein-coding genes (PCGs), two ribosomal RNAs (rRNAs), 22 transfer RNAs (tRNAs), and a putative control region. Its gene content and organization were identical to those of the closely related species, *E. ohtai*, and they showed 67.2% identity for the whole mitogenome.

All PCGs had an ATN start codon except ND4L which started with GTG. Also, most of the PCGs terminated with a complete stop codon (TAA or TAG) while COX3 and ND4 had an incomplete stop codon (T-). The lengths of 16S and 12S rRNAs were 1,284 bp (70.6% AT content) and 757 bp (66.2% AT content), respectively. The size of tRNA genes ranged from 61 to 70 bp. A putative control region (309 bp; 68.2% AT content) was located between the 12S rRNA and tRNA^Lys^.

Phylogenetic trees were constructed with maximum likelihood (ML) and Bayesian inference (BI) methods using 13 PCGs of 19 barnacles (Figure 1). The tree topology of the ML and BI methods were congruent. Based on the phylogenetic tree, *E. ohtai* and *E. coreana* were monophyletic with high supporting values (100% bootstrap proportions and 1.00 Bayesian posterior probabilities). In addition, the genus *Eochionelasmus* was separated far from another vent barnacle *Vulcanolepas fijiensis*, further suggesting two independent origins of hydrothermal vent barnacles in agreement with previous studies (Herrera et al. 2015; Chan et al. 2019).

**Figure 1.**
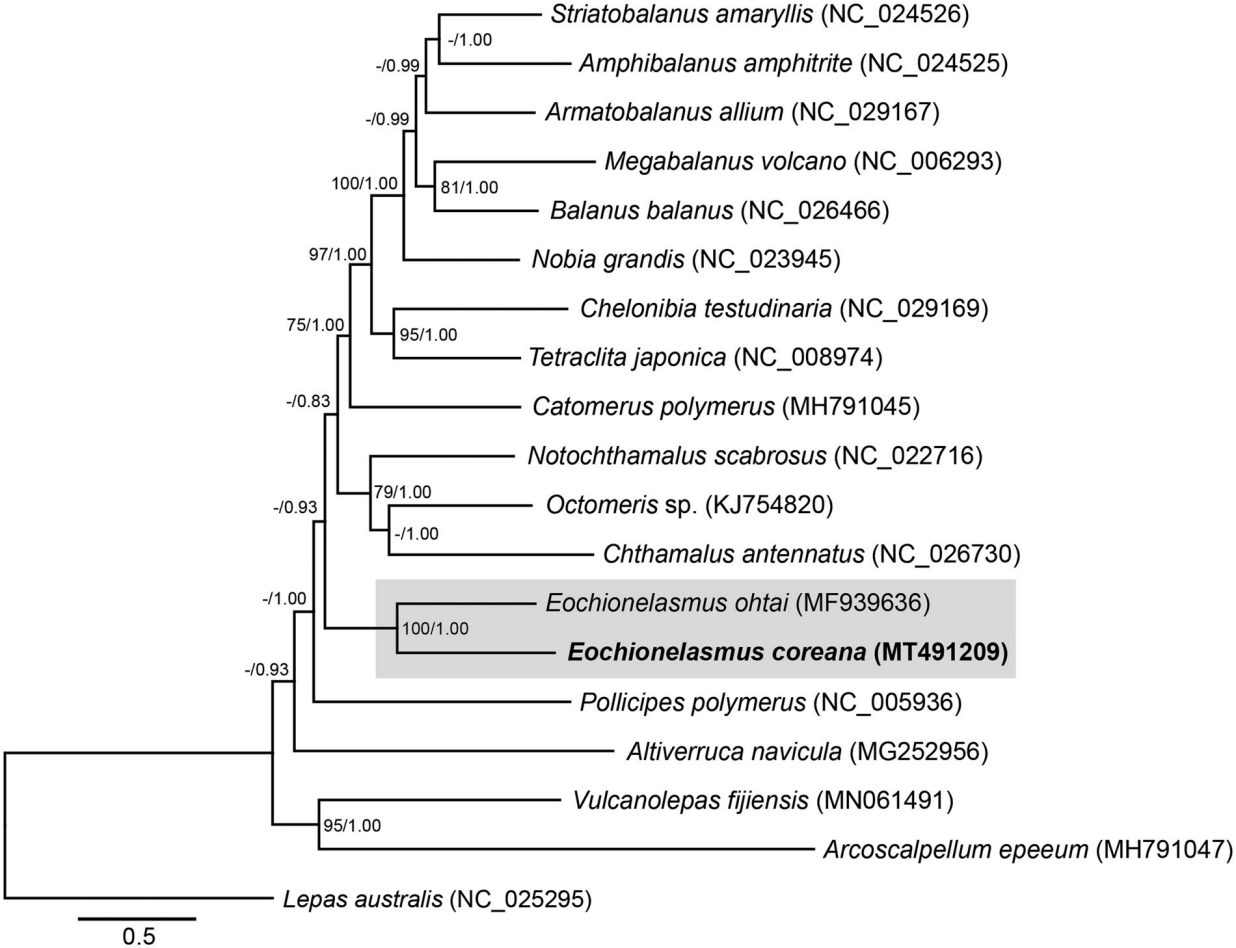
Phylogenetic tree of *Eochionelasmus coreana* and other barnacles based on 13 mitochondrial protein-coding genes using maximum-likelihood (ML) and Bayesian inference (BI) methods. *Eochionelasmus* species are emphasized with a gray shade box. GTR + G was selected as the best evolutionary model using jModelTest 2.1.4. Numbers on internodes indicate maximum likelihood bootstrap proportions (left) and Bayesian posterior probabilities (right). Hyphen (-) indicates a bootstrap value less than 60%.

It has been known that non-coding regions on mitogenomes are informative occasionally for solving taxonomic and phylogenetic problems (Place et al. 2005). In case of the genus *Eochionelasmus*, a long intergenic region between tRNA^Pro^ and tRNA^Thr^ in mitogenome of *E. coreana* could be the key to deepen our understanding about the speciation and global distribution of *Eochionelasmus* species. Accordingly, further mitogenomic analysis of the last *Eochionelasmus* species, *E. paquensis*, is required.

## Data Availability

The data that support the findings of this study are openly available in GenBank at [https://www.ncbi.nlm.nih.gov/genbank/], reference number MT491209.
